# Impact of disrespectful maternity care on childbirth complications: a multicentre cross-sectional study in Ethiopia

**DOI:** 10.1186/s12884-024-06574-0

**Published:** 2024-05-21

**Authors:** Ephrem Yohannes, Gonfa Moti, Gemechu Gelan, Debra K. Creedy, Laura Gabriel, Carolyn Hastie

**Affiliations:** 1 School of Nursing and Midwifery, Griffith University, University Drive, Gold Coast, QLD 4131 Australia; 2 Midwifery Department, College of Health Sciences and Referral Hospital, Ambo University, Ambo, Ethiopia; 3 Surgery Department, College of Health Sciences and Referral Hospital, Ambo University, Ambo, Ethiopia

**Keywords:** Central Ethiopia, Childbirth related complications, Cross-sectional, Disrespectful maternity care

## Abstract

**Background:**

Globally, disrespectful, and abusive childbirth practices negatively impact women’s health, create barriers to accessing health facilities, and contribute to poor birth experiences and adverse outcomes for both mothers and newborns. However, the degree to which disrespectful maternity care is associated with complications during childbirth is poorly understood, particularly in Ethiopia.

**Aim:**

To determine the extent to which disrespectful maternity care is associated with maternal and neonatal-related complications in central Ethiopia.

**Methods:**

A multicentre cross-sectional study was conducted in the West Shewa Zone of Oromia, Ethiopia. The sample size was determined using the single population proportion formula. Participants (*n* = 440) were selected with a simple random sampling technique using computer-generated random numbers. Data were collected through face-to-face interviews with a pretested questionnaire and were entered into Epidata and subsequently exported to STATA version 17 for the final analysis. Analyses included descriptive statistics and binary logistic regression, with a 95% confidence interval (CI) and an odds ratio (OR) of 0.05. Co-founders were controlled by adjusting for maternal sociodemographic characteristics. The primary exposure was disrespectful maternity care; the main outcomes were maternal and neonatal-related complications.

**Results:**

Disrespectful maternity care was reported by 344 women (78.2%) [95% CI: 74–82]. Complications were recorded in one-third of mothers (33.4%) and neonates (30%). Disrespectful maternity care was significantly associated with maternal (AOR = 2.22, 95% CI: 1.29, 3.8) and neonatal-related complications (AOR = 2.78, 95% CI: 1.54, 5.04).

**Conclusion:**

The World Health Organization advocates respectful maternal care during facility-based childbirth to improve the quality of care and outcomes. However, the findings of this study indicated high mistreatment and abuse during childbirth in central Ethiopia and a significant association between such mistreatment and the occurrence of both maternal and neonatal complications during childbirth. Therefore, healthcare professionals ought to prioritise respectful maternity care to achieve improved birth outcomes and alleviate mistreatment and abuse within the healthcare sector.

## Introduction

Maternal and neonatal mortality rates remain high in several low- and middle-income countries, despite substantial reductions over the past two decades [1]. The Sustainable Development Goals target a global average of 70 maternal deaths per 100,000 live births and a national goal of fewer than 140 maternal deaths per 100,000 live births by 2030 [2]. In Ethiopia, the overall number of maternal deaths declined by 55% from 31,000 in 1990 to 14,000 in 2017 [3]. However, Ethiopia still ranks fourth and sixth globally in terms of maternal and neonatal fatalities, respectively [4, 5]. Advocating for institutional births and upholding respectful maternal care are crucial strategies for reducing both maternal and newborn mortality rates, especially in low-income countries [6].

The latest strategic plan for Ethiopia’s healthcare sector prioritizes compassionate and respectful maternity care as one of its four main goals for transformation [7]. The formulation of this plan was informed by the recognition of inadequate care and mistreatment as major obstacles to the utilization of facility-based delivery services [8]. The proportion of home births is estimated to range between 39.62% and 81.45% across various regions [9]. Women who choose home births often cite experiences or fears of disrespect and abuse during facility-based childbirth as factors influencing their decision [10]. Studies conducted in various nations have demonstrated that when women are made to feel esteemed, respected, and at ease, they are more inclined to make use of healthcare services, voice their concerns, and undergo preventative or curative measures at an early stage [11].

The issue of “obstetric violence and mistreatment” has garnered significant attention and is widely recognized as a serious breach of human rights [12]. The term “obstetric violence” refers specifically to the infliction of physical, sexual, and/or verbal abuse, coercion, bullying, humiliation, and/or assault by medical professionals, including nurses, doctors, and midwives, upon women during the course of labour and delivery [13]. The present research utilized the World Health Organization’s (WHO) definition of “disrespect and abuse during facility-based childbirth” [14], which condemns physical, verbal, and disrespectful childbirth practices and emphasizes women’s rights to respectful care, life, health, and freedom from discrimination [14].

Obstetric mistreatment and abuse are particularly evident in low-income countries where women are marginalised and oppressed [15]. Disrespectful maternity care can manifest as treatment denial, disregard for the needs and pain of the patient, intrusive behaviour, medication overuse, forced medical intervention, detention in facilities due to nonpayment, rude or dehumanising treatment, and discrimination or humiliation based on age, gender nonconformity, race, ethnicity, or economic status, among other factors [14, 16]. Examples of disrespectful care include repeated vaginal examinations carried out by multiple healthcare professionals; perineal repairs performed without anaesthesia; uterine investigations; verbal, physical, and sexual assault; extortion; imprisonment; and invasion of privacy [17].

The frequency of mistreatment during childbirth in Ethiopia has been widely documented. A systematic review and meta-analysis conducted recently reported that the overall prevalence of disrespect and abuse in maternal health care during labour and birth was 49.4% [18]. In a 2017 study conducted across four regions of Ethiopia in various healthcare facilities, it was found that 36% of women had experienced at least one form of mistreatment during childbirth [19]. Nevertheless, recent reports have indicated that the prevalence of obstetric violence among Ethiopian mothers spans from 76.1 to 98.9% [20].

Despite the prevalence of disrespectful and abusive care in Ethiopia, the impact on birth outcomes remains under-researched [21]. Researchers have also suggested investigating the negative effects of disrespect and abuse of women on neonatal outcomes [22]. It is crucial to conduct evidence-based research to develop and evaluate interventions that prevent disrespect and abuse, thereby protecting women’s rights, enhancing the quality of care, increasing demand for facility-based births, and contributing to improved health outcomes. This study aims to determine the associations between disrespectful maternity care and adverse birth outcomes in the central region of Ethiopia.

## Methods

### Design, setting and study population

A cross-sectional study was conducted in the West Shewa Zone of Oromia in Ethiopia, which has a population of 2,058,676 people and a total of 134 health facilities, including eight hospitals, 92 health centres, and 520 health posts. Four hospitals and fifteen health centres that had a higher volume of births were selected for the study. All medical facilities in the region offer labour and birth services [23]. In this region, it is common for mothers who give birth at home to attend a healthcare institution during the early postpartum period for a range of reasons, such as treating infections or birth complications, receiving routine postnatal care, getting immunisations, and using family planning services. However, the childbirth-related complications experienced by this group of mothers might not be due to the disrespect and abuse behaviours of health care providers during postpartum care. Therefore, this study recruited only low-risk, postpartum women who attended a healthcare facility, regardless of the mode of their childbirth.

### Sample size calculation and sampling procedure

The sample size was determined using the single population proportion formula with the assumption that 49.9% (P) of women in Ethiopia experienced disrespect and abuse [18] at a 5% level of significance and 5% margin of error. The total sample size required was 442 after considering a 15% non-response rate. The total sample size was distributed proportionally to each site based on quarterly reports of birth rates. Women’s medical registration numbers were used to collect data via simple random sampling with a computer-generated random number approach.

### Variables and measures

The study utilised the ‘Mother on Respect Index’ (MORI) scale due to its reliability and validity in low-income countries [24]. This scale has 14 items describing respectful or disrespectful care to which women respond on a yes/no basis. Women were deemed to have received disrespectful maternity care if they affirmed any form of disrespect [24].

A woman is typically considered to have experienced a childbirth complication if any of the following is recorded: perineal tear, maternal mortality, maternal anxiety, postpartum haemorrhage, postpartum depression, puerperal sepsis, uterine inversion, uterine rupture, preterm labour, prolonged labour, or obstructed labour. Additionally, neonatal complications may include stillbirth, preterm birth, a low APGAR score, foetal distress, an abnormal heartbeat, umbilical bleeding, neonatal birth injuries, and neonatal sepsis [25]. Several of these complications were identified through review of the medical registration book, while additional complications were determined via a survey of mothers who had recently given birth.

### Data collection tools and procedures

Data were gathered between January 1st and February 30th, 2023. A pre-tested, standardized questionnaire was administered through face-to-face interviews. The data was collected immediately following discharge from the postnatal clinic in a separate location. Moreover, certain relevant data were extracted from the women’s medical report logbook. Both the consent forms and questionnaire were written in English and translated to Afan Oromo. The tool included disrespectful maternity care details (the 14 items of MORI scale) [24] and 38 items developed from the literature related to sociodemographic characteristics, antenatal care, and labour, childbirth related complications, and postpartum experiences [25, 26]. Eight individuals were recruited and trained as data collectors, none of whom were employed at a participating facility. Three individuals with Master’s degrees oversaw the data collectors. The researchers provided a two-day training program that covered the study’s purpose, ethical and interpersonal considerations, and data collection and storage procedures. The questionnaire was pretested on 22 women who gave birth at a participating site, with the aim of identifying any ambiguity, ensuring consistency, and making any necessary revisions. Daily, the supervisors or lead investigators collected the completed surveys and reviewed them for completeness.

### Data processing and analysis

The data from the survey was refined, encoded, and inputted into the Epidata software program version 3.1, which was subsequently exported to STATA version 17 for further analysis. The analysis comprised descriptive statistics and binary logistic regression with a 95% confidence interval (CI) and an odds ratio (OR) of 0.05. The assessment of multicollinearity was conducted, and it was determined that there was no presence of multicollinearity. The confounding factors were managed by restricting the respondents to those with low-risk pregnancies.

## Results

### Sociodemographic characteristics

Four hundred forty women completed the questionnaire, yielding a 99% response rate. The mean age was 28.38 years (SD ± 6.46), ranging from 17 to 39 years. Almost all women (*n* = 419, 95.2%) were married. Most women (*n* = 320, 72.7%) and their husbands (*n* = 316, 71.8%) attended primary school and above (detailed information is presented in Table 1).

**Table 1:** Sociodemographic characteristics of women (*n* = 440)

Variables	Category	Frequency *n* (%)
Age	< 25	193 (43.9%)
	25–29	95 (21.6%)
	30–34	129 (29.3%)
	≥ 34	23 (5.2%)
Religion	Muslim	107 (24.3%)
	Waqefata	76 (17.3%)
	Protestant	257 (58.4%)
Educational status	No formal education	120 (27.3%)
	Primary school and above	320 (72.7%)
Husband’s educational status	No Formal education	124 (28.2%)
	Primary school and above	316 (71.8%)
Marital status	Married	419 (95.2%)
	Separated	8(1.8%)
	Divorced	6 (1.4%)
	Widowed	7 (1.6%)
Women’s occupation	Farmer	116 (26.4%)
	Merchant	48 (10.9%)
	Government employee	21 (4.8%)
	Housewife	255 (58%)
Distance from health facility	Within a radius of 10 km or equal	52 (11.8%)
	Greater than a radius of 10 km	388 (88.2%)

### Obstetric-related characteristics

Most respondents (*n* = 280, 63.6%) were multiparous, and 296 (67.3%) had no antenatal clinic (ANC) care during pregnancy. Of the total respondents, 298 (67.7%) gave birth by spontaneous vaginal delivery, and 53.9% gave birth at hospital. Women who gave birth at home during their previous pregnancy (*n* = 117, 26.6%) were predominantly assisted by traditional birth attendants. (detailed information is presented in Table 2).

**Table 2:** Obstetric-related characteristics of women (*n* = 440)

Variables	Category	Frequency *n* (%)
Parity	Primipara	160(36.4%)
	Multipara	280(63.6%)
Place of birth of the previous pregnancy	Home	193(43.9%)
	Health Facility (Includes current pregnancy)	247(56.1%)
Reason for previous home birth	Insufficient information about health facility birthing services	7(1.6%)
	My previous birth was normal/no problem	6(1.4%)
	My husband wants me to give birth at home	8(1.8%)
	Financial constraints	17(3.9%)
	Lack of transportation	52(11.8%)
	Fear of procedures	28(6.4%)
	Fear of Covid-19 pandemic	14(3.2%)
	No telephone service to call the ambulance	25(5.7%)
	Labour was too short	36(8.2%)
Who assisted the childbirth at home?	Mother	22(5%)
	Traditional birth attendant	117(26.6%)
	Healthcare provider	8(1.8%)
	Health extension worker	41(9.3%)
	Emergency assistant working with the Ambulance	5(1.1%)
Did you have an ANC care for this pregnancy?	Yes	144(32.7%)
	No	296(67.3%)
When did you start ANC in the current pregnancy?	Before/at 16wks	48(10.9%)
	After 16wks	96(21.8%)
How many ANC contacts did you have?	One	21(4.8%)
	Two	68(15.5%)
	Three	26(5.9%)
	Four or more	29(6.6%)
Place of birth for this pregnancy	Hospital	237(53.9%)
	Health centre	203(46.1)
Mode of current birth	SVB	298(67.7%)
	C/S	81(18.4%)
	Instrumental delivery	61(13.9%)

### Maternal and neonatal-related complications during childbirth

Most respondents (*n* = 341, 77.5%) reported that they had encountered at least one type of complication during childbirth. There were 147 (33.4%) cases of unfavourable neonatal-related birth outcomes. The overall maternal-related complication rate during and after childbirth was 30% (*n* = 132) (for detail information refer to Table 3).

**Table 3:** Maternal and neonatal-related complications

Maternal Related Variables	Present	Frequency (%)
Prolonged labour	No	411(93.4%)
	Yes	29(6.6%)
Obstructed labour	No	385(87.5%)
	Yes	55(12.5%)
Premature rupture of membrane	No	421(95.7%)
	Yes	19(4.3%)
Uterine rupture	No	434(98.6%)
	Yes	6(1.4%)
Uterine inversion	No	432(98.2%)
	Yes	8(1.8%)
Perineal injury	No	416(94.5%)
	Yes	24(5.5%)
Post-partum haemorrhage	No	361(82%)
	Yes	79(18%)
Puerperal sepsis	No	399(90.7%)
	Yes	41(9.3%)
Post-partum depression	No	416(94.5%)
	Yes	24(5.5%)
Maternal anxiety	No	434(98.6%)
	Yes	6(1.4%)
Maternal mortality	No	435(98.8%)
	Yes	5(1.2%)
**Neonatal Related variables**		
Preterm	No	411(93.4%)
	Yes	29(6.6%)
Foetal distress	No	365(83%)
	Yes	75(17%)
Abnormal heartbeat	No	394(89.5%)
	Yes	46(10.5%)
Stillbirth	No	422(95.9%)
	Yes	18(4.1%)
Umbilical bleeding	No	412(93.6%)
	Yes	28(6.4%)
Hypothermia	No	420(95.4%)
	Yes	20(4.6%)
Low APGAR score	No	363(82.5%)
	Yes	77(17.5%)
Neonatal infection	No	371(84.3%)
	Yes	69(15.7%)
Neonatal birth injuries	No	352(80%)
	Yes	88(20%)
Admitted to NICU	No	374(85%)
	Yes	66(15%)

### Prevalence of disrespectful maternity care

The overall proportion of disrespectful maternity care during childbirth was 344 (78.2%) (95% CI: 74–82). According to birth location, 47% of women who gave birth at health centres, 68.8% who gave birth at hospitals, and 72.3% who gave birth at home in their previous pregnancy (visit health institution for routine postnatal care) received disrespectful maternity care (RMC) (*p* < 0.001). Among mothers reporting disrespectful care, most (81.8%) felt uneasy while receiving care, 63% reported experiencing verbal or physical abuse, and 54% believed their care was indecent (details are presented in Table 4).

**Table 4:** Women’s reports of disrespectful maternity care (*n* = 440)

Variables	Present	Frequency (%)
I felt comfortable asking question	No	291(66.1%)
	Yes	149(33.9%)
I felt comfortable declining the care that was offered	No	277(63%)
	Yes	163(37%)
I felt comfortable accepting the options for care that my doctor or midwife recommended	No	269(61.1%)
	Yes	171(38.9%)
I felt pushed into accepting the options my doctor or midwife suggested	Yes	283(64.3%)
	No	157(35.7%)
I chose the care options that I received	No	263(59.8%)
	Yes	177(40.2%)
My personal preferences were respected	No	239(54.3%)
	Yes	201(45.7%)
My cultural preferences were respected	No	247(56.1%)
	Yes	193(43.9%)
I was treated poorly because of my race, ethnicity, cultural background, or language	Yes	269(61.1%)
	No	171(38.9%)
I was treated poorly because of my sexual orientation	Yes	276(62.7%)
	No	164(37.3%)
I was treated poorly because of my level of private health insurance	Yes	265(60.2%)
	No	175(39.8%)
I was treated poorly because of a difference of opinion with my caregivers about the right care for myself or my baby	Yes	249(56.6%)
	No	191(43.4%)
I held back from asking questions or discussing my concerns because my care provider seemed rushed	Yes	271(61.6%)
	No	169(38.4%)
I held back from asking questions or discussing my concerns because I wanted maternity care that differed from what my care provider recommended	Yes	274(62.3%)
	No	166(37.7%)
I held back from asking questions or discussing my concerns because I thought my care provider might think I was being difficult	Yes	262(59.5%)
	No	178(40.5%)

### Associations between disrespectful maternity care and neonatal and maternal-related complications

Neonatal-related complications were noted in 36.9% (*n* = 127) of mothers who reported disrespectful treatment. A maternal-related complication was reported by 34% (*n* = 117) of mothers who were treated disrespectfully. Respondents who reported disrespectful maternity care were two times more likely to experience neonatal-related complications than were those who received respectful care [AOR = 2.22, 95% CI: 1.29, 3.8; *p* < 0.004]. Participants who encountered disrespectful care during childbirth were almost three times more likely to experience a maternal-related complication than were those who had respectful care [AOR = 2.78, 95% CI: 1.54, 5.04; *p* < 0.001].

## Discussion

The research findings indicate that disrespectful maternity care is alarmingly common in the central area of Ethiopia. In general, a third of mothers and newborns experienced birth-related difficulties. It was discovered that disrespectful maternity care was significantly connected to adverse birth outcomes. Approximately three-quarters of respondents in this study (78.2%) reported being treated disrespectfully, which is comparable to the outcomes of similar research conducted in Addis Abeba (79%) the capital city of Ethiopia [27]. The high prevalence of disrespectful maternity care may be attributed to Ethiopia’s maternity care model, which primarily focuses on medical procedures rather than woman-centred approaches [28]. The prevalence of disrespectful maternity care in this study was greater than that reported in studies performed in other regions (Tigray, Amhara, Oromia and Southern Nations, Nationalities and People of Ethiopia) (36%) [19] and northern Ethiopia (22%) [26]. The current study revealed that 63% of mothers experienced physical or verbal abuse, 81.8% felt discomfort during care, and 54% received undignified care. In comparison, a study performed in another region indicated that 40.5% of women received non-dignified care, 22.2% reported abuses, and 30.1% reported inappropriate cultural consideration [20].

Fluctuations in the level of respect accorded to women may be attributable to the proportions and methods of care provided by healthcare professionals, including limited knowledge and unfavourable attitudes towards RMC, normalisation of disrespect and mistreatment, and insufficient adherence to guidelines [8, 29]. Moreover, variations in healthcare settings and systems, such as high workloads, inadequate infrastructure, and insufficient workforce capacity building, may exacerbate staff stress and contribute to women’s disregard. Variations in research factors may also impact the findings. Differences in socioeconomic and cultural characteristics among participants across various studies, the timing of data collection (immediately after birth versus postpartum), different sampling techniques, and the way disrespect and abuse are defined and measured may affect outcomes. Some studies involved observing practices, while others utilised standardised self-report tools such as the MORI. It is conceivable that observers familiar with the healthcare system may not perceive certain practices as disrespectful, whereas asking for women directly may reveal significant differences [19]. Future studies should prioritize additional research aimed at eliminating observer effects, as this will be crucial in evaluating changes in practice.

Although the prevalence of disrespectful care in the current study was lower than the extent of mistreatment and abuse reported in other low-income countries, such as 98% in Nigeria [30], 99.7% in Pakistan [31] and 97.4% in Peru [32], it is still a concern that needs to be addressed. It appears that the Ethiopian government’s efforts to prioritise respectful maternity care are beginning to bear fruit for women giving birth. The gap in the incidence of respectful maternity care between Ethiopia and other countries may be attributed to several variables, including differences in methodology, study duration, socioeconomic status, health policy, facilities, culture, and infrastructure [33]. Moreover, Ethiopia has made significant strides in increasing the proportion of births in healthcare facilities, thereby placing additional pressure on the healthcare system to provide high-quality obstetric care [34]. Ethiopia appears to have experienced less disrespectful care than other countries mentioned above, which may be attributable to several factors.

A prior Ethiopian study found that women with pregnancy and childbirth challenges were more likely to endure mistreatment, whereas those who underwent a caesarean birth following a vaginal birth experienced less mistreatment. This study also discovered a connection between disrespectful maternity care and neonatal and maternal complications during childbirth [33]. A study demonstrated that a higher number of occurrences of disrespect and mistreatment were correlated with a less favourable birth experience. Over 90% of women who did not encounter any such incidents reported a positive birth experience. However, the positive experience rate decreased by 10–15% with each additional instance of disrespect or mistreatment [35]. While it is not our intention to promote the excessive use of obstetric intervention, when necessary, caesarean sections can save lives, and women may perceive their emergency care as respectful, less painful, and result in positive neonatal outcomes.

Previous research has shown that mistreatment and abuse in childbirth are associated with various negative maternal and newborn health consequences, including postpartum bleeding, physical injuries, and emotional issues like post-traumatic stress disorder and suicidal thoughts [36, 37]. Inappropriate behaviour during childbirth can lead to significant psychological distress, including nightmares, panic, fear of childbirth, demoralization, and difficulty forming a bond with the child. Additionally, it can have a detrimental impact on emotional and sexual relationships [38, 39]. For instance, in Nigeria, postpartum women disclosed that verbal abuse and yelling were prevalent in the birthing suite and intensified their challenges during childbirth [40]. Furthermore, some women hold the view that the healthcare system is responsible for childbirth-related issues due to the provision of subpar care [41]. Research has demonstrated that mistreatment and abuse during labour can have detrimental consequences on the initiation of early breastfeeding, which in turn increases the likelihood of neonatal morbidity and mortality [42]. Furthermore, a history of abusive care and low expectations can create a lack of trust in the healthcare system and influence future decisions regarding giving birth in health facilities, particularly in low-income and middle-income countries [15].

The utilization of facility-based childbirth and postnatal health care can be hindered by negative experiences during childbirth, which serve as impediments. This aversion can have far-reaching consequences for both maternal health and the health of newborns [43]. Respectful maternity care is an essential element for a positive childbirth experience; however, disrespectful, and abusive treatment can adversely affect women’s decision to give birth in institutional settings. Various factors contribute to disrespectful care, such as complications, instrumental deliveries, care provided in government hospitals, and low wealth index. Women who have experienced complications are six times more likely to be disrespected and abused during childbirth, whereas those who undergo instrumental deliveries are 2.7 times more likely to face disrespect and abuse compared to those who have spontaneous vaginal deliveries [44, 45]. Research highlights the importance of addressing disrespectful treatment and providing respectful care to all women, regardless of their country of origin, mode of birth and place of birth. Although certain women opt for home births due to cultural appropriateness and supportive care, others prioritize medical interventions. Those who choose home births may prefer the liberty to move around, avoid surgical procedures, and evade physical restraints. Consequently, this study indicates that fulfilling the desires of women who prefer to give birth at home is feasible in healthcare institutions if Respectful Maternity Care is fully implemented [46]. Planned home birth can provide a sense of comfort and involve fewer interventions, but it may also be linked to substandard care and complications resulting from insufficient resources for managing potential complications [47].

In summary, it is essential to address disrespectful treatment of childbirth women to achieve favourable birth results and safeguard the rights of women during childbirth. The ill-treatment that occurs during childbirth gives rise to detrimental consequences, both physical and emotional, for both the mother and the newborn, and has long-lasting implications for the family’s emotional and relational well-being. Therefore, it is imperative to enhance the quality of maternal health care.

### Limitations

This study had several limitations. While the MORI tool is commonly used in low- and middle-income countries, some of its items reflect poor communication or inattentive behaviour rather than “disrespectful maternity care”. To address this, it is necessary to develop tools that explicitly target abusive behaviour, accurately reflect women’s desired care experiences, and assist staff in improving their approach to care. Our survey did not cover all significant complications associated with childbirth, such as abnormal presentations, abnormal positions, abnormal amniotic fluid levels, and placenta-related complications. Furthermore, it is essential to recognize the cross-sectional design of the study limitations when implementing the findings of this research. It is essential to recognise that the association between disrespectful maternity care and childbirth complications is not cause-and-effect. The present study may have faced limitations due to women’s reluctance to report suboptimal care while still receiving postpartum care. To address this issue, the researchers made every effort to reassure the participants about their anonymity and collected data in a confidential location. However, the results of the current study may not be generalisable to all women, as only those with low-risk pregnancies were included. Future studies should focus on translating current knowledge into practice and addressing the evidence and performance gaps related to the implementation of respectful maternity care (RMC) to reduce disrespectful care. Qualitative, randomised controlled trials (RCTs), observational, and mixed-method studies are better suited for exploring these issues in the future. Furthermore, the role of fathers/partners in decision-making regarding birth choices and care during labour and birth warrants further investigation.

## Conclusion

Despite the World Health Organization’s desire for every woman to receive respectful and dignified medical treatment, disrespectful care continues to be a public health problem in Ethiopia. The current research conducted in the study area found that high levels of disrespectful maternity care exist. This research demonstrated a significant connection between disrespectful maternity care and both maternal and neonatal complications during childbirth. It is essential for healthcare providers to prioritise respectful maternity care as a primary objective, to improve birth outcomes and prevent harassment and mistreatment within the healthcare system. Stakeholders, particularly women and healthcare providers, ought to collaborate to enhance the quality of care and eradicate disrespectful and abusive behaviours within healthcare establishments.

## Data Availability

The corresponding author will provide the datasets used and/or analyzed during the current work upon request.

## Abbreviations

ANC: Antenatal Care
C/S: Caesarean section
NICU: Neonatal intensive care unit
RMC: Respectful Maternity Care
SVB: Spontaneous vaginal birth
WHO: World Health Organization
